# The Werther effect following the suicides of three korean celebrities (2017–2018): an ecological time-series study

**DOI:** 10.1186/s12889-023-16080-1

**Published:** 2023-06-19

**Authors:** Li-Hyun Kim, Gyeong-Min Lee, Woo-Ri Lee, Ki-Bong Yoo

**Affiliations:** 1 Department of Healthcare Institution Support, National Health Insurance Service, Wonju, Republic of Korea; 2 Department of premedical, College of Medicine, Dankook University, Cheonan, Republic of Korea; 3 Department of Resarch and Analysis, National Health Insurance Service Ilsan Hospital, Goyang, Republic of Korea; 4 Division of Health Administration, College of Software and Digital Healthcare Convergence, Yonsei University, Wonju, Republic of Korea

**Keywords:** Suicide, Werther effect, Idol singer, Media reporting, Suicide reporting guidelines

## Abstract

**Background:**

The suicide rate in Korea was the highest among the member countries of the Organization for Economic Cooperation and Development(OECD) for 2013–2016 and 2018–2020. In korea, suicide was the leading cause of death among individuals aged 10–39, and the second leading cause of death for aged 40–59. Thus, this study aimed to examine the Werther effect of the suicides of three Korean idol singers (Jonghyun: December 18, 2017, Sulli: October 14, 2019, and Hara Gu: November 24, 2019).

**Methods:**

The study conducted Poisson regression and used the cause-of-death statistics microdata from 2016 to 2020 provided by Statistics Korea. The case periods ranged from the day of the suicide of each celebrity to 10 weeks after. The control periods were all weeks from 2016 to 2020, excluding the case periods.

**Results:**

The suicide rates in Korea significantly increased by 1.21, 1.30, and 1.28 times after the deaths of Jonghyun, Sulli, and Hara Gu, respectively. The Werther effect was more evident in women than men. Suicide rate among individuals aged 10–29 years was greater than those for other age groups.

**Conclusions:**

This study confirmed that the rate of copycat suicides increased after three celebrity singers in Korea died by suicide. Nevertheless, the rate of suicide after the suicide of the three celebrity singers was lower than those in previous studies in Korea.

## Background

Suicide is a serious social problem worldwide. According to Statistics Korea, the number of suicides per 100,000 people in Korea as of 2020 was 25.7 [1], which was more than double the average suicide rate of 11.0 reported by the Organization for Economic Cooperation and Development (OECD) among its member countries. The suicide rate in Korea was the highest among OECD member countries for 2003–2020 [2]. In 2020, suicide was the leading cause of death among individuals aged 10–39, and second leading cause of death for 40–59[1]. Specifically, 41.1%, 54.4%, and 39.4% of the causes of deaths of individuals aged in their 10s, 20 and 30 s, respectively, were due to suicide [1].

The Werther effect is a phenomenon in which cases of suicide increase after the publication of suicide news due to imitation. For this reason, it is typically called *copycat suicide*. Phillips first used the term in 1974, which is based on Goethe’s novel entitled *The Sorrows of Young Werther*. In the novel, the main character called Werther died by suicide, and many people imitated his death. Phillip demonstrated that the number of suicides in the United States increased after a newspaper reported a suicide case. In other words, the more the publicity given to a case of suicide, the higher the number of suicides that follow [3].

Celebrity suicides are widely reported in the media, which leads to a significant impact on subsequent suicides [4, 5]. Niederkrotenthaler [4] conducted a systematic review and meta-analysis and found that suicide risk increased by 13% after media reports of suicide by celebrities, and deaths in this context increased by 30% after media reports of the suicide methods used by celebrities. Similarly, Stack collected 293 results from 42 studies through a systematic literature review. The results demonstrated that the studies on the Werther effect of well-known celebrities were 14.3 times more likely to reveal a copycat effect [5]. In a study that analyzed the relationship between 109 celebrity suicides and number of daily suicides in Japan from 1989 to 2010, the number of suicide increased by an average of 5.5% over 10 days after a media reporting of celebrity suicides [6]. According to Chen [7], suicide risk has increased by 1.17 times across two weeks after the suicide of 24-year-old female singer Ivy Li. Studies in Korea have also examined the Werther effect. The risk of suicide increased by 73% during the first week following the suicides of actress Jin-sil Choi, in which the Werther effect lasted for six weeks [8]. Ha analyzed changes in the number of suicides within 10 days after the suicide of 13 celebrities, which statistically significantly increased [9].

Recognizing the Werther effect, the World Health Organization (WHO) stated that the “responsible reporting of suicide” is one of the three major policies for suicide prevention [10]. Furthermore, the Korean government also recognized the Werther effect; thus, the Ministry of Health and Welfare, the Central Suicide Prevention Center, and the Korea Journalists Association established Suicide Reporting Guidelines in July 2004 and subsequently revised it in September 2013 and July 2018.

Suicide Reporting Guidelines 3.0 consists of five principles. First, expressions, such as *death* and *die*, should be used in the titles of media news instead of *suicide*. Second, reporters should not disclose specific suicide methods, tools, motives, and locations. Third, photos or videos related to suicide should be used with care. Fourth, the negative consequences of suicide and suicide prevention information should be cited, and suicide should not be not glorified or rationalized. Fifth, the personality of the deceased should be respected, and the privacy of the bereaved family should be protected [11]. The guidelines mentioned that compliance should include Internet broadcasting, single-person broadcasting, and social networking services (SNSs) [11].

Recently, three singers in Korea died by suicide, namely, Jonghyun (December 18, 2017), Sulli (October 14, 2019), and Hara Gu (November 24, 2019). They diagnosed with depression during their lifetime. They were singers who gained immense popularity among the youth. Teenagers and those aged in their 20s watch the performances and activities of these singers through various media, thus forming an attachment to them [12]. A number of the youth buy products advertised by these singers and wish to be identified with them. Moreover, they purchase hundreds of compact discs to win fan meetings and provide expensive gifts to the singers.

After establishing Suicide Reporting Guidelines 3.0 (July 31, 2018), 88.5% of the newspaper articles no longer mentioned suicide methods [13]. Similarly, after the suicides of Sulli and Hara Gu, 99.6% of the media did not mention the suicide methods when reporting their death [14]. This rate was higher than that of a study (33.3%) on guideline compliance rates in 2005 [15]. As a result, the magnitude of the impact of celebrity suicides on the public has changed in Korea due to the revision of and increased compliance with the guidelines. However, studies on the Werther effect have been lacking since the establishment of Suicide Reporting Guidelines 3.0 in Korea.

Prior to Jonghyun’s suicide, there had been no reported suicides by idol singers in Korea. These celebrities gain popularity and exert a large influence on the youth; thus, their suicide may exert a larger impact on the youth. For this reason, this study discussed the Werther effect and its implications after the suicide of the three celebrities using cause-of-death statistics data from 2016 to 2020 in Korea.

## Methods

### Study design and data

We used the microdata of the cause-of-death statistics from 2016 to 2020 provided by Statistics Korea to examine the Werther effect observed following the suicides of the three Korean singers. Cause-of-death statistics are aggregated according to the Korean Standard Classification of Diseases (KCD) by analyzing the causes of death of deceased individuals on the basis of reports received by each region under the Statistics Act and the Family Register Act [16]. Microdata pertain to individual unit data and are provided without personal identity.

The case periods ranged from the day of the suicide of each celebrity to 10 weeks after (Jonghyun [Case 1]: December 18, 2017–February 25, 2018, Sulli [Case 2]: October 14, 2019–December 22, 2019; Hara Gu [Case 3]: November 24, 2019–February 1, 2020). The control periods were all weeks from 2016 to 2020, excluding the case periods. We set 10 weeks as a case period to consider long-term effects. Korea observes a Buddhist funeral culture called Sasipgu-jae or the Forty-Nine Day Ceremony (49 Jae), in which prayers for peace for departed ones are offered every seven days across for 49 days. Memorial messages are posted, and memorial services are held on 49 Jae; thus, the study assumed that the Werther effect may extend beyond this ceremony.

### Variables

The dependent variable was the number of suicides per week. We extracted data coded as X60–X84 according to the KCD classification of the cause-of-death. Extracted data were calculated based on the day of the suicide of each celebrity.

We added meteorological and economic factors as control variables, which were previously found associated with suicide [17–22]. Moreover, we used temperature, humidity, sunshine duration, seasonal variations, unemployment rate, and composite coincident index as control variables. The composite coincident index is a composite estimate of the current trend of the economy. It is estimated using indexes for manufacturing production, service industry activity, and retail sales [23]. Temperature, humidity, and sunshine duration were entered into the model by calculating the average values per week, while seasonal variations were controlled for by designating winter as the reference point. Unemployment rate and component coincident index are measured per month; thus, we entered the same value per month. Data on meteorological factors were collected from the Korea Meteorological Administration [24], and economic factors were collected from the Korean Statistical Information Service [25, 26].

### Statistical analysis

The study used Poisson regression to estimate changes in suicide rate after the suicide of the celebrities. Poisson regression is used when a dependent variable is a rare event that occurs within a specific range such as suicide [27]. The Poisson regression model for estimating changes in suicide rates after the suicide of the three celebrities is as follows:

$$\begin{aligned} &\log(u_{t}) = \upbeta_{0} + \upbeta_{1} \times Week_{t} + \upbeta_{2} \times Temperature_{t} + \upbeta_{3} \times Humidity_{t} + \upbeta_{4} \times\\ &Duration\ of\ Sunshine_{t} \text{-} \sum\nolimits^{4}_{i=1} \upbeta_{i} \times \text{season i} + \upbeta_{5} \times Unemployment\ Rate_{t} + \upbeta_{6} \times\\ &\text{Cyclical Component of Coincident index}_{t} + \mathrm{e}_{t}, \end{aligned}$$

$\mu_t$; number of suicides per week;

: week after celebrity suicide (1–10); other periods (0);

Seasons 1–4: indicators of seasonal reflection (0,1); and

: error term.

Week is the major independent variable that represents the suicide of each singer. This variable enabled the study to examine the changes in relative rate of suicide after the suicide of the celebrities in the Poisson regression model. The seasons were used as the categorical variables, and temperature, humidity, sunshine duration, unemployment rate, and component coincident index were presented as the continuous variables.

## Results

**Fig. 1:**
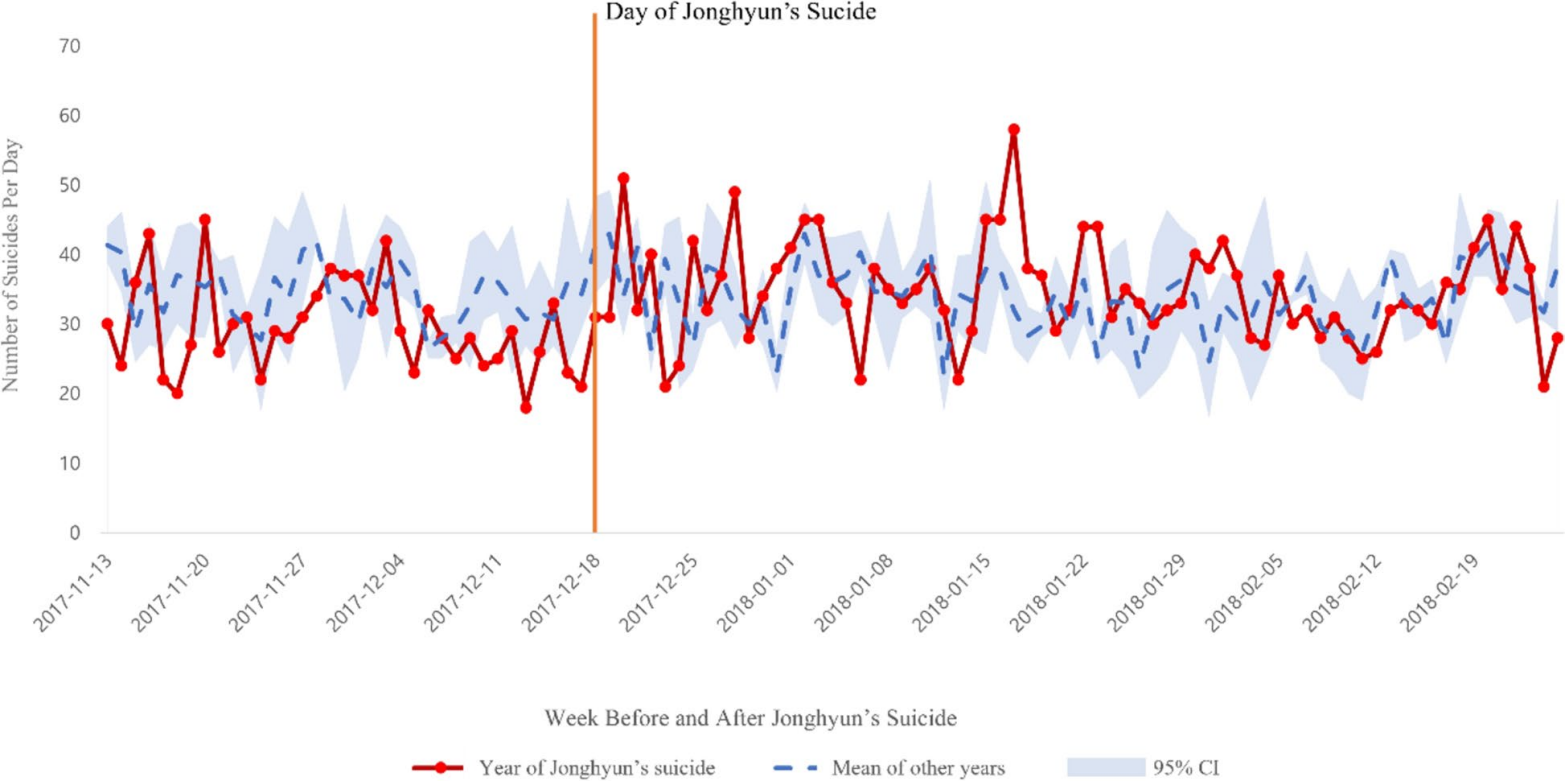
Changes in the number of suicides before and after idol Jonghyun’s suicide

**Fig. 2:**
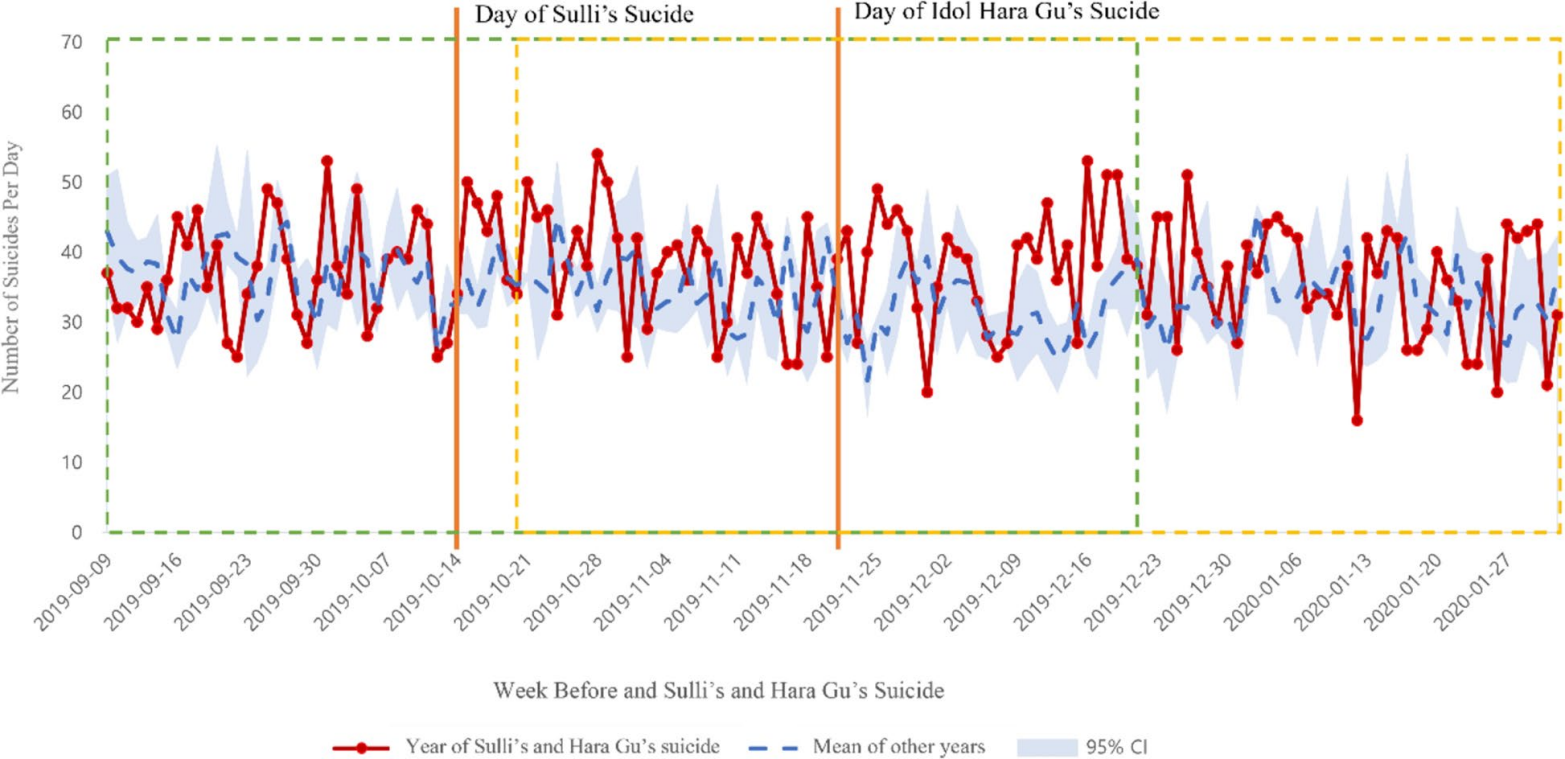
Changes in the number of suicides before and after Sulli’s and Hara Gu’s suicide

We compared the number of suicides in the 10 weeks (70 days) after the suicide of each of the celebrities and mean of the number of suicides within the same period in other years (Figs. 1 and 2). After Jonghyun’s suicide, the number of suicides in weeks 2, 5–7, and 10 was higher than those in other periods. Moreover, after Sulli’s suicide, the number of suicides within 1–10 weeks was higher than those in corresponding periods in other years. Similarly, after Hara Gu’s suicide, the number of suicides in weeks 1–7 was higher than that in other years.

Although the increased number of total suicides after Jonghyun’s suicide was statistically non-significant (*p* = 0.407), those after Sulli’s and Hara Gu’s suicide statistically significantly increased (Table 1). After Sulli’s suicide, the number of suicides for men and women has increased. However, only the increase in suicides among women has been significant (*p* < 0.001), with more than twice the number of suicides compared to men (12 vs. 23). After Hara Gu’s suicide, suicide by both men and women significantly increased (Table 1). A statistically significant increase in suicides was observed among individuals aged 10–29 years after the suicides of the three celebrities. After Sulli’s suicide, suicides among those aged 10–29 and 30–49 years increased by 15. Moreover, suicides increased by 12 and 8 among individuals aged 10–29 and 30–49 years, respectively, after Hara Gu’s suicide (Table 1).

**Table 1:** Changes in the number of suicides in 10 weeks following three idol singers

	Year of Death	Other Years	*t*-value	*p*-value
Jonghyun (Suicide on December 18, 2017)				
Total Suicide	243.90 ± 21.82	235.50 ± 28.82	0.84	0.407
Male	176.60 ± 13.06	167.90 ± 21.20	1.22	0.229
Female	67.30 ± 13.39	67.67± 10.88	−0.09	0.931
10 to 29 Years of Age	30.10±5.61	25.63±6.19	2.02	0.051
30 to 49 Years of Age	91.4±012.69	85.63±11.55	1.34	0.190
50 to 69 Years of Age	82.00±9.25	81.90±13.00	0.02	0.982
70 Years Of Age Or Older	40.40±4.81	42.37±9.48	−0.85	0.400
Sulli (Suicide on October 14, 2019)				
Total Suicide	269.50 ±21.81	235.00 ±26.53	3.79	0.004
Male	178.10 ±13.47	166.60 ±20.15	1.71	0.093
Female	91.40±12.69	68.45±10.76	5.82	<0.001
10 to 29 Years of Age	42.20±8.24	26.95±8.16	5.27	<0.001
30 to 49 Years of Age	91.90±9.62	77.05±10.41	4.09	<0.001
50 to 69 Years of Age	86.30±12.21	81.55±12.91	1.05	0.298
70 Years Of Age Or Older	49.00±6.38	49.43±9.74	−0.13	0.897
Hara Gu (Suicide on November 24, 2019)				
Total Suicide	257.70 ±22.23	229.10 ±25.05	3.20	0.003
Male	178.90 ± 13.71	165.00 ± 18.56	2.17	0.034
Female	78.80±10.54	64.10±9.27	4.20	<0.001
10 to 29 Years of Age	35.80±7.73	24.22±5.45	5.41	<0.001
30 to 49 Years of Age	90.70±8.59	82.33±13.90	1.80	0.078
50 to 69 Years of Age	84.90±11.53	77.08±11.96	1.84	0.072
70 Years Of Age Or Older	46.20±9.21	40.53±9.51	1.68	0.100

*CI *Confidence interval, *RR *Relative rate

Table 2 presents the Poisson regression results related to the suicides of the three celebrities. The rate of suicide increased significantly in weeks 2–3 and 5–6 after Jonghyun’s suicide. The rate of suicide was the highest in the fifth week after Jonghyun’s suicide, which was 1.21 times more than that observed in the corresponding period. After Sulli’s suicide, the suicide rate significantly increased in weeks 1–2 and 9–10. The relative rates of suicide in the first and second weeks were 1.17 and 1.18, respectively. Suicide rate in the 10th week after Sulli’s suicide was the highest with a relative rate of 1.30. After Hara Gu’s suicide, suicide rate increased significantly in weeks 3–5. Suicide rates in the weeks 3, 5, and 5 were 1.19, 1.28, and 1.19, respectively, which was the highest at week 4 with a relative rate of 1.28. The suicide rate in spring and fall, particularly in spring, was higher than that in winter. When temperature increased by 1 °C, relative suicide rate increased by 0.2%. The humidity increased by 1%, relative rate increased by 0.2-0.4%. The effect of sunshine duration on suicide was statistically non-significant. As unemployment rate increased by 1%, relative suicide rate increased by 3.8–4.7%. Lastly, as the composite index of business indicators increased by one unit, relative suicide rate increased by 0.3–0.4%.

**Table 2:** Changes in the relative rate of suicide associated with three idol singers

		Jonghyun		Sulli		Hara Gu	
		**RR**	**95% CI**	**RR**	**95% CI**	**RR**	**95% CI**
Periods	**Other Periods**	1.000					
	**Week 1**	1.018	(0.892 − 1.162)	1.165	(1.036–1.309)	1.115	(0.988–1.258)
	**Week 2**	1.191	(1.052 − 1.349)	1.175	(1.046–1.320)	1.064	(0.935–1.210)
	**Week 3**	1.180	(1.043 − 1.336)	1.124	(0.997–1.267)	1.190	(1.053–1.344)
	**Week 4**	0.984	(0.861–1.125)	1.042	(0.919–1.180)	1.283	(1.141–1.442)
	**Week 5**	1.211	(1.076–1.364)	1.016	(0.895–1.154)	1.186	(1.050 − 1.339)
	**Week 6**	1.157	(1.020–1.314)	1.057	(0.933–1.198)	1.108	(0.980 − 1.254)
	**Week 7**	1.082	(0.952–1.229)	1.101	(0.975–1.245)	1.055	(0.930 − 1.197)
	**Week 8**	0.935	(0.816–1.073)	1.036	(0.909–1.181)	1.003	(0.880 − 1.143)
	**Week 9**	1.000	(0.874–1.145)	1.185	(1.049–1.338)	0.948	(0.830 − 1.083)
	**Week 10**	1.111	(0.978–1.262)	1.303	(1.160–1.463)	1.013	(0.891–1.151)
Seasons	**Winter**	1.000					
	**Spring**	1.137	(1.101–1.174)	1.126	(1.090–1.162)	1.145	(1.109 − 1.183)
	**Summer**	1.043	(0.995–1.094)	1.039	(0.990 − 1.090)	1.059	(1.009 − 1.112)
	**Autumn**	1.057	(1.021–1.096)	1.049	(1.012–1.088)	1.065	(1.026 − 1.105)
Temperature		1.001	(0.999 − 1.004)	1.002	(1.000–1.005)	1.002	(1.000 − 1.005)
Humidity		1.004	(1.001–1.006)	1.003	(1.001–1.005)	1.002	(1.000–1.004)
Duration of Sunshine		1.007	(0.999–1.015)	1.004	(0.996–1.011)	0.999	(0.992–1.007)
Unemployment Rate		1.038	(1.015–1.062)	1.047	(1.024–1.070)	1.039	(1.017–1.062)
Composite Index of Business Indicators		1.004	(1.002–1.007)	1.003	(1.001–1.005)	1.004	(1.001–1.006)

*CI *Confidence interval, *RR *Relative rate

Table 3 presents the results of gender stratification analysis. After Jonghyun’s suicide, suicide rate significantly increased in weeks 2, 5, 6, and 10 for men and weeks 3, 5, and 7 for women. Moreover, women were at higher rate of copycat suicide after the suicide of the female celebrity, which, thus significantly increased after Sulli’s suicide at weeks 1–3, 6–7, and 9–10. However, no significant increase in suicide rate was observed for men. For Sulli, suicide rate was the highest at week 2 with a 50% increase compared with that of the control period. For Hara Gu, a significant increase in suicide was observed in week 4 for men, while an increase in suicide rate was observed in weeks 3–5. Suicide rates for women were 1.34, 1.39, and 1.31 in weeks 3, 4, and 5, respectively.

**Table 3:** Changes in relative rate of suicide associated with the three idol singers by gender

		Jonghyun				Sulli				Hara Gu			
		**Male**		**Female**		**Male**		**Female**		**Male**		**Female**	
		**RR**	**95% CI**	**RR**	**95% CI**	**RR**	**95% CI**	**RR**	**95% CI**	**RR**	**95% CI**	**RR**	**95% CI**
Periods	**Others**	1.000				1.000				1.000			
	**Week 1**	1.046	(0.896–1.221)	0.951	(0.739–1.224)	1.082	(0.937–1.250)	1.361	(1.115–1.662)	1.079	(0.932–1.250)	1.197	(0.968–1.480)
	**Week 2**	1.200	(1.035–1.390)	1.172	(0.930–1.478)	1.037	(0.895– 1.202)	1.503	(1.242– 1.818)	1.045	(0.896–1.219)	1.112	(0.878–1.408)
	**Week 3**	1.147	(0.988–1.331)	1.262	(1.011–1.576)	1.006	(0.865–1.169)	1.407	(1.154–1.715)	1.130	(0.974–1.310)	1.340	(1.080–1.662)
	**Week 4**	1.043	(0.89–1.218)	0.841	(0.644–1.098)	0.997	(0.856–1.160)	1.149	(0.923–1.431)	1.240	(1.077–1.428)	1.391	(1.127–1.718)
	**Week 5**	1.185	(1.027–1.367)	1.276	(1.029–1.582)	1.000	(0.859– 1.164)	1.056	(0.840– 1.327)	1.137	(0.982–1.317)	1.308	(1.052–1.625)
	**Week 6**	1.196	(1.031– 1.388)	1.064	(0.834–1.359)	0.939	(0.802–1.100)	1.339	(1.090– 1.645)	1.068	(0.920–1.239)	1.210	(0.970–1.509)
	**Week 7**	1.010	(0.864–1.181)	1.258	(1.009–1.569)	1.025	(0.881–1.192)	1.283	(1.041–1.581)	1.009	(0.866–1.175)	1.169	(0.935–1.463)
	**Week 8**	0.971	(0.828–1.139)	0.847	(0.648–1.108)	1.030	(0.882–1.203)	1.052	(0.825–1.341)	1.005	(0.861–1.173)	1.000	(0.782–1.277)
	**Week 9**	1.108	(0.952–1.290)	0.733	(0.547–0.981)	1.107	(0.954–1.284)	1.378	(1.115–1.702)	0.915	(0.779–1.073)	1.032	(0.813–1.311)
	**Week 10**	1.186	(1.025–1.373)	0.925	(0.713–1.199)	1.258	(1.094– 1.447)	1.415	( 1.149–1.742)	1.060	(0.913–1.230)	0.896	(0.694–1.155)
Season	**Winter**	1.000			1.000					1.000			
	**Spring**	1.137	(1.094–1.181)	1.136	(1.070–1.206)	1.126	(1.084–1.169)	1.126	(1.061–1.195)	1.142	(1.099–1.187)	1.153	(1.085–1.225)
	**Summer**	1.023	(0.967–1.082)	1.093	(1.002–1.192)	1.021	(0.964–1.081)	1.084	(0.992–1.184)	1.040	(0.982–1.102)	1.107	(1.013–1.211)
	**Autumn**	1.041	(0.998–1.086)	1.097	(1.028–1.171)	1.036	(0.992–1.082)	1.081	(1.010 − 1.157)	1.039	(0.994–1.085)	1.132	(1.057–1.212)
Temperature		1.002	(0.999–1.005)	1.000	(0.996–1.005)	1.002	(0.999–1.005)	1.002	(0.998–1.007)	1.003	(0.999–1.006)	1.001	(0.997–1.006)
Humidity		1.004	(1.001–1.006)	1.004	(1.000–1.008)	1.003	(1.000–1.005)	1.003	(0.999–1.007)	1.002	(0.999–1.004)	1.003 33	(0.999–1.007)
Duration of Sunshine		1.007	(0.997–1.016)	1.006	(0.991–1.021)	1.004	(0.995–1.014)	1.002	(0.987–1.017)	0.998	(0.989–1.007)	1.003	(0.988–1.017)
Unemployment Rate		1.046	(1.019–1.074)	1.020	(0.978–1.062)	1.047	(1.020–1.075)	1.046	(1.004–1.090)	1.038	(1.012–1.065)	1.041	(1.000–1.084)
Composite Index of Business Indicators		1.003	(1.000–1.006)	1.007	(1.003–1.012)	1.003	(1.000–1.005)	1.004	(1.000–1.009)	1.003	(1.000–1.006)	1.005	(1.001–1.010)

*CI *Confidence interval, *RR *Relative rate

Table 4 presents the results of the statistical analysis according to age stratification. The rate of copycat suicide was higher among those aged 10–29 years compared with the other age groups. After Jonghyun, the study observed no statistically significant increase in suicide rate in the majority of weeks among those aged > 50 years. However, a significant increase in suicide rate was noted for those aged 10–29 and 30–49 years. Among those aged 10–29 years, suicide rates increased by 43.9% and 44.8% in weeks 2 and 3, respectively. In addition, among those aged 30–49 years, suicide rate increased by 24.5–40.9%. After Sulli’s suicide, suicide rate among those aged 10–29 years significantly increased. Especially, suicide rate among those aged 10–29 years increased more than twice in week 1. Suicide rate among those aged 10–29 years increased by up to 62% and 53% in weeks 2 and 3, respectively. Lastly, after Hara Gu, suicide rate increased by 46.6–88.8% in weeks 1–3 among those aged 10–29 years. Furthermore, an increase in suicide rate was observed among those aged 30–49 years in weeks 1 and 4.

**Table 4:** Changes in relative rate of suicide by age associated with three idol singers

		Jonghyun				Sulli				Hara Gu			
		**10 to 29** **Years of Age** **RR**	**30 to 49** **Years of Age** **RR**	**50 to 69** **Years of Age** **RR**	**70 Years of Age or Older** **RR**	**10 to 29** **Years of Age** **RR**	**30 to 49** **Years of Age** **RR**	**50 to 69** **Years of Age** **RR**	**70 Years of Age or Older** **RR**	**10 to 29** **Years of Age** **RR**	**30 to 49** **Years of Age** **RR**	**50 to 69** **Years of Age** **RR**	**70 Years of Age or Older** **RR**
Periods	**Other Periods**	1.000 1.000 1.000											
	**Week 1**	1.254	0.938	1.096	0.890	2.096^*^	1.100	1.095	0.891	1.466^*^	1.247^*^	1.016	0.863
	**Week 2**	1.439^*^	1.290^*^	1.162	0.912	1.622^*^	1.207	1.158	0.919	1.888^*^	1.001	0.877	0.994
	**Week 3**	1.448^*^	1.409^*^	0.987	0.933	1.530^*^	1.269^*^	0.867	1.110	1.570^*^	1.052	1.021	1.565^*^
	**Week 4**	0.988	1.100	0.920	0.840	1.354	0.961	1.082	0.935	1.375	1.268^*^	1.251^*^	1.316
	**Week 5**	1.330	1.245^*^	1.156	1.174	1.089	1.112	0.984	0.885	1.118	1.137	1.159	1.386^*^
	**Week 6**	1.217	1.048	1.311^*^	1.055	1.134	1.291^*^	0.891	0.930	1.197	1.003	1.140	1.195
	**Week 7**	1.219	1.042	1.099	1.039	1.561^*^	1.140	1.086	0.799	1.109	1.041	0.927	1.309
	**Week 8**	0.687	0.927	1.012	0.966	1.858^*^	0.953	0.824	1.071	1.007	1.139	0.951	0.814
	**Week 9**	1.306	1.026	0.869	1.014	1.584^*^	1.074	1.038	1.454^*^	1.271	1.029	0.810	0.838
	**Week 10**	0.993	1.183	1.109	1.068	1.450^*^	1.255^*^	1.256^*^	1.403^*^	0.869	0.898	1.101	1.164
Season	**Winter**	1.000 1.000 1.000											
	**Spring**	1.187^*^	1.076^*^	1.164^*^	1.179^*^	1.153^*^	1.051	1.157^*^	1.205^*^	1.194^*^	1.054	1.187^*^	1.224^*^
	**Summer**	1.163^*^	1.016	0.986	1.140^*^	1.142	0.989	0.986	1.183^*^	1.160^*^	1.000	1.017	1.199^*^
	**Autumn**	1.141^*^	0.987	1.040	1.180^*^	1.112	0.956	1.033	1.233^*^	1.145^*^	0.971	1.052	1.234^*^
Temperature		1.000	0.995	1.003	1.010^*^	1.005	0.997	1.003	1.010^*^	1.000	0.997	1.003	1.012^*^
Humidity		1.002	1.007^*^	1.004	0.999	0.998	1.006^*^	1.003	0.999	1.003	1.005^*^	1.002	0.997
Duration of Sunshine		1.002	1.016^*^	1.004	0.999	0.990	1.012	1.004	0.997	1.003	1.011	0.995	0.986
Unemployment Rate		1.051	1.029	1.050^*^	1.028	1.101^*^	1.033	1.046^*^	1.043	1.067^*^	1.030	1.040^*^	1.039
Composite Index of Business Indicators		1.025^*^	1.000	1.007^*^	0.996	1.019^*^	0.999	1.007^*^	0.995	1.021^*^	0.999	1.008^*^	0.995^*^

*RR *Relative rate; ^*^P < 0.05

## Discussion

The study aimed to examined the Werther effect observed following the suicides of three Korean celebrity singers. The results demonstrated significant increases in suicide rates after the their deaths. This finding is consistent with those of previous studies on the Werther effect, which pointed to increases in suicides after celebrity suicides [4, 8, 9, 28]. However, the magnitude was smaller than those of previous studies [8, 9]. This result may be related to compliance with the principles of the revised Suicide Reporting Guidelines in Korea, which subsequently increased after its implementation. Especially, compliance rate with the principle of refraining from mentioning suicide methods in detail significantly increased. In a study conducted in 2005, which is prior to the formulation of Suicide Reporting Guidelines, only 33.3% of the media refrained from mentioning suicide methods [15]. However, this rate increased to 88.5% in another study conducted in 2022, which was after the release of Suicide Reporting Guidelines 3.0 [14]. A study conducted in Austria supported the current finding in that the number of suicides in Austria decreased after the establishment of media guidelines. Specifically, the Crisis Intervention Center Vienna introduced media guidelines and asked newspapers to adhere to the guidelines and monitor the articles. Afterward, a reduction of 81 suicides per year was noted. Particularly, suicide reduction was significantly higher in regions with high rates of compliance compared with those of other regions (− 47.48 vs. −0.24) [29].

The current findings demonstrated that women were at higher rate of copycat suicide after the suicide of the three celebrities. This result was not consistent with those of previous studies, which proposed that the rate of copycat suicide in men was high after the suicide of male celebrities and that the rate of copycat suicide in women was high after the suicide of female celebrities. Another study found that copycat suicide was higher in women after the suicides of Korean actress Jin-sil Choi and Taiwanese female singer Ivy Li [7, 8]. Moreover, the rate of copycat suicide following that of American actor Robin Williams was higher in men [30]. Jonghyun was a celebrity singer with numerous female fans; thus, the rate of copycat suicide by women may have been relatively high. According to a report by Melon, which is the top music streaming site in Korea, 83% of the fans of SHINee (the group to which Jonghyun belongs) were women. Thus, the rate of copycat suicide may vary according to coverage, circumstances, and characteristics at the time of a celebrity suicide [31]. The urge to suicide can become stronger if people are interested in the celebrities who died due to suicide. Immediately after Jonghyun, an incident occurred in Indonesia in which one fan mimicked Jonghyun’s suicide while mourning his death [32].

Our findings also showed that the rate of copycat suicide was higher among individuals aged 10–29 years compared with those of other age groups. This finding was consistent with that of a previous study that demonstrated an increase in suicide among women aged 30–39 years after the suicide of the Korean actress Jin-sil Choi (age: 39 years) [8]. Horizontal identification and vertical identification explain the reason for the high rate of copycat suicide among women in this age group. Horizontal identification, that is, individuals tend to identify themselves with others who are similar with their demographic characteristics such as gender, age and social position. Vertical identification, on the other hand, argues that individuals identify themselves with others who are famous, popular, admired and perceived as superior [5, 33]. Three celebrities who were less than 30 years old and gained tremendous popularity among the younger generation. Thus, individuals aged 10–29 years may feel connection and strengthen identify with the three celebrities and mimic their behaviors. Suicide-related harmful information in SNSs, such as Facebook and Instagram, also appeared to influence the high rate of copycat suicide among those aged 10–29 years. In 2018, 82.3% and 73.3% of people aged in their 20 and 30 s, respectively, used SNS, which was higher than the rates of use in other age groups (55.9%, 39.6%, and 18.9% for those aged in their 40s, 50s, and 60s, respectively) [34]. The Ministry of Health and Welfare, the National Police Agency, and the Central Suicide Prevention Center intensively monitored information on the internet, such as photos, videos, suicide methods and a companion suicide that could influence suicide-related behaviors from July 18–31. They found 17,338 cases of suicide-related information, and 77% cases from SNS [35]. People aged 10–29 years were more likely to be exposed to suicide-related harmful information that do not adhere to the guidelines, which could strengthen emotional identification.

The decrease in the rate of copycat suicide after the establishment of Suicide Reporting Guidelines 3.0 suggests the importance of compliance with the guidelines. Despite the tendency of indiscriminate media reports on suicide to lead to copycat suicides, more news in Internet portals, online video platforms, and SNSs did not comply with the guidelines than did newspapers. In Korea, the percentages of people who watched the news through Internet portals, online video platforms (e.g., YouTube), and SNSs (e.g., Facebook and Instagram) were 79.2%, 69.7%, and 45.4%, respectively. Only 8.9% of people read the news via newspapers [36]. Moreover, the rate of copycat suicide was higher for television, which provides information accompanied by visual and auditory materials than those for newspapers and radios [37]. Despite the high rate of copycat suicide due to the abovementioned platforms, media constantly produce sensational contents related to suicide.

Thus, exerting effort to increase compliance with Suicide Reporting Guidelines 3.0 is necessary for types of media. All media and individuals who make contents should be given the social responsibility to comply with the guidelines. Moreover, the government should establish the policies to increase compliance rates. As such, cooperation within the media industry is one of the methods for increasing compliance with the guidelines. For example, in Austria, the media closely followed the guidelines on suicide reporting, which resulted in a reduction in suicide. In Australia, organizations were directly contacted during guideline dissemination, which led to high levels of awareness of the guidelines [38]. However, China’s guideline of not cooperating with the media appeared to be ineffective in reducing suicide [39]. In Korea, the Ministry of Health and Welfare, the Central Suicide Prevention Center, and the Korea Journalists Association jointly revised the Suicide Reporting Guidelines. Moreover, journalists were involved in formulating the guidelines to increase compliance [11]. Although many people watch the news through Internet portals or online video platforms, cooperation and distribution within the media remain insufficient. Therefore, the government needs to actively cooperate in the establishment and distribution of the guidelines with the overall media industry as well as journalist associations to increase compliance.

The limitations of this study are as follows: First, ecological errors may emerge when the conclusion from an observed group is applied to individual cases [40]. This result only demonstrated that the suicides of the three celebrities were associated with increases in suicide rates among the Korean population. Whether or not the deaths of the three celebrities influenced the subsequent cases of suicide should be analyzed through studies at the individual level. However, the results of the current study could be used to inform suicide prevention policies that target the overall community. Second, the study overlooked other variables, such as medical history and socioeconomic levels. Previous studies demonstrated that economic level, mental illness, cancer, and the presence of chronic diseases are related to suicide [4]. Therefore, future studies should consider these variables.

## Conclusion

The study confirmed that suicide rate significantly increased after the suicides of the three celebrities. Increase in suicide rate among women was higher than men, and increase in suicide rate among individuals aged 10–29 years was higher than other age groups. However, the magnitude of suicide rate increase was smaller than those observed in previous studies conducted before the implementation of Suicide Reporting Guidelines 3.0. Despite the importance of compliance with the guidelines, a significant portion of content in Internet portals, online video platforms, and SNSs did not comply with the guidelines. Compliance with guidelines in all forms of media should be emphasized as one of the strategies for suicide prevention. All forms of media and content producers should recognize that their suicide reporting could increase copycat suicide and comply with the guidelines. Moreover, the government should cooperate with all forms of media to revise and promotion the guidelines, in order to increase compliance.

## Data Availability

The datasets generated and analyzed during the current study are publicly available in the MicroData Integrated Service (https://mdis.kostat.go.kr/index.do).

## Abbreviations

OECD: Organization for Economic Cooperation and Development
WHO: World Health Organization
KCD: Korean Standard Classification of Diseases
SNS: Social Networking Services
